# Cardiovascular biomarkers and risk of low-energy fractures among middle-aged men and women—A population-based study

**DOI:** 10.1371/journal.pone.0203692

**Published:** 2018-09-14

**Authors:** Maria Härstedt, Anna Holmberg, Cecilia Rogmark, Richard Sutton, Olle Melander, Viktor Hamrefors, Artur Fedorowski

**Affiliations:** 1 Department of Clinical Sciences, Faculty of Medicine, Lund University, Malmö, Sweden; 2 Department of Paediatrics, Skåne University Hospital, Malmö, Sweden; 3 Department of Orthopaedics, Skåne University Hospital, Malmö, Sweden; 4 National Heart and Lung Institute, Imperial College, Hammersmith Hospital Campus, London, United Kingdom; 5 Department of Internal Medicine, Skåne University Hospital, Malmö, Sweden; 6 Department of Cardiology, Skåne University Hospital, Malmö, Sweden; Medical College of Wisconsin, UNITED STATES

## Abstract

**Background:**

Low-energy fractures are a growing health challenge as their incidence increases with advancing age. As cardiovascular instability may be associated with higher likelihood of traumatic falls, we aimed to investigate the associations between four cardiovascular biomarkers and the risk of low-energy fractures in a middle-aged population.

**Methods:**

A total of 5291 individuals from the prospective Malmö Diet and Cancer (MDC) study (mean age, 57 years; 59% women) with data on baseline levels of four cardiovascular biomarkers: mid-regional-fragment of pro-adrenomedullin-peptide (MR-pro-ADM), mid-regional-fragment of pro-atrial-natriuretic-peptide (MR-proANP), N-terminal pro-brain natriuretic peptide (NT-pro-BNP) and C-terminal-pro-arginine-vasopressin (CT-pro-AVP/Copeptin) were included. The associations between biomarker levels and first incident low-energy fracture were tested in Cox proportional-hazard models, taking potential interactions and traditional risk factors into account.

**Results:**

Participants were followed for a median time of 21.0 years, during which 1002 subjects (19%) experienced at least one low-energy fracture. Subjects with incident fracture were older, more likely to be women, had lower BMI and higher prevalence of previous fractures. Among biomarkers, there was a significant interaction between gender and MR-pro-ADM on the risk of fracture (p = 0.002). MR-pro-ADM predicted fractures in men only (hazard ratio, 1.23; 95% CI 1.09–1.40; p = 0.001), whereas there was no association among women. Levels of MR-pro-ANP, NT-pro-BNP and CT-pro-AVP did not predict fractures.

**Conclusions:**

Higher circulating levels of MR-pro-ADM predict low-energy fractures among middle-aged-men, whereas levels of MR-pro-ANP, NT-pro-BNP and CT-pro-AVP are not associated with increased fracture risk. Further controlled studies should test the hypothesis whether MR-pro-ADM may improve prediction of bone fractures.

## Introduction

Low-energy fractures are defined according to WHO as fractures resulting from standing height or less, also defined as fragility fractures that result from mechanical forces that would not ordinarily result in a fracture [1]. Fragility fractures occur most commonly in the spine (vertebrae), hip (proximal femur) and wrist (distal radius) but may also occur in the arm (humerus), pelvis, ribs and other bones. These fractures are a growing health challenge in the developed countries as their incidence increases with advancing age of the population. The problem is expected to expand as the number of the elderly living to a very old age will be higher in the decades to come[2]. Consequently, there is a need for better strategies aimed at the identification of high-risk patients, in whom more intensive preventive interventions should be directed[2].

Low-energy fractures constitute more than 90% of all fractures and usually follow low-energy falls[2]. Among various factors that may contribute to falls among the elderly are orthostatic instability (imbalance and hypotension) which may lead to sudden loss of body control [3]. Low-energy fractures are more common among women than men, mainly due to the genetic and environmental effect of osteoporosis, which affects women to a higher degree [4]. Following low-energy fractures, especially hip fractures, excess mortality is high in the first year and is higher in older men than in women[5].

We have previously shown that orthostatic hypotension and higher heart rate predict low-energy fractures in the population [6, 7]. Several studies have shown that cardiovascular and cerebrovascular comorbidities are common among patients suffering from low-energy fractures[4, 5]. Moreover, we have shown that some cardiovascular biomarkers, such as copeptin (CT-pro-AVP), pro-atrial-natriuretic-peptide (MR-pro-ANP), and pro-adrenomedullin (MR-pro-ADM) may be altered in conditions related to unexplained falls and syncope such as orthostatic hypotension [8, 9]. Given the strong evidence of correlation between cardiovascular instability and traumatic falls and injuries, it is appealing to explore how markers of increased cardiovascular risk may relate to the incidence of low-energy fractures.

We aimed to investigate the association between four established cardiovascular biomarkers and the incidence of low-energy fractures in a middle-aged population.

## Methods

### Study population

As previously described[10–12], the Malmö Diet and Cancer (MDC) study is a population-based, prospective epidemiologic cohort of approximately 30 000 persons of Caucasian race enrolled between 1991 and 1996. The subjects were born 1926–1945 (age, 44–74 years at inclusion). The main goal of the MDC was to study the impact of diet on cancer incidence and mortality. The study consisted of a baseline examination including dietary assessment, a self-administered questionnaire including smoking status, alcohol consumption and medical history, anthropometric measurements and blood pressure, using a mercury sphygmomanometer and appropriate-size cuff, after 10 minutes supine resting. Current smoking was defined as smoking within the past year. Hypertension was defined as blood pressure exceeding 140/90 mmHg or the use of antihypertensive medication.

A random 50% of enrolled subjects in MDC were also invited to take part in a study of cardiovascular risk factors. This sub-cohort, which is referred to as the MDC Cardiovascular Cohort (MDC-CC) consists of 6103 subjects (60% women) [11]. Of these, 5540 subjects also agreed to additional blood tests provided under standardized fasting conditions. In the years 2009–2010, several cardiovascular (CV) biomarkers were measured and prospectively associated with the risk of incident CV disease and diabetes in this subset of original MDC-CC cohort[10, 11]. For the aims of current study, subjects with successful measurement of at least one of the four cardiovascular biomarkers: MR-pro-ADM, MR-proANP, CT-pro-AVP, and N-terminal pro-brain natriuretic peptide (NT-pro-BNP) were included, meaning that 5291 subjects were eligible for inclusion. We hypothesized that biomarkers associated with higher probability of orthostatic hypotension might predict fall-related fragility fractures. All participants provided written informed consent and the MDC study was approved by the ethical committee at Lund University, Lund, Sweden.

### Analysis of biomarkers in MDC

The circulating fragments of the cardiovascular markers were measured from fasting blood samples that had been frozen at -80° C after collection at MDC baseline. Levels of MR-pro-ADM, MR-pro-ANP and CT-pro-AVP were measured using immunoluminometric sandwich assay targeted against amino acids in the respective peptide according to the manufacturer’s instructions: Thermo Scientific BRAHMS KRYPTOR (BRAHMS GmbH, part of Thermo Fisher Scientific, Hennigsdorf, Germany)[13–15]. Levels of NT-pro-BNP were determined using the Dimension RxL automated N-BNP method (Siemens Diagnostics, Nürnberg, Germany)[16].

Mean inter-assay coefficients of variation were ≤10% for MR-proANP and MR-proADM, 2.7% for NT-pro-BNP [11] and <20% for the assay for CT-pro-AVP as previously described [15]

### Definition and retrieval of incident low-energy fractures

The primary endpoint in the study was first incident low-energy fracture during follow-up. Information about low-energy fracture diagnoses was requested from the Swedish National Patient Register (SNPR) and covered the period from MDC baseline through Dec 31, 2014. Low-energy fractures were defined according to International Classification of Diseases (ICD), 10^th^ Revision. In this study we included low-energy fractures affecting the spine and the thoracic cage (M48.x, M84.x, M96.x, S12.x and S22.x), the upper extremities including arms, shoulders and hands (S42.x, S52.x and S62.x), the pelvis (S32.x), as well as the hips and femur (S72.x).

### Statistical analyses

Group differences in continuous variables between low-energy fracture -positive and -negative individuals were compared using Student’s T-test, whereas categorical variables were compared using Pearson’s chi-square test. Levels of three of the four biomarkers, MR-pro-ANP, NT-pro-BNP and CT-pro-AVP, were log-transformed in all analyses due to skew deviation. MR-pro-ADM was normally distributed. The associations between levels of biomarkers at baseline and first incident fragility fracture during follow-up were tested in Cox proportional hazard models. The time variable was calculated in years from baseline to the end of follow-up on December 31, 2014 or death, emigration, or the date of first incident fracture, whichever occurred first. We first used minimally (age and gender) adjusted models. If significant associations were found for one of the biomarkers we additionally tested the relation in models adjusted for smoking, systolic blood pressure, antihypertensive medication, BMI and previous low-energy-fracture, alcohol consumption (denoted as below or over median consumption in the cohort) and prevalent CVD at baseline (defined as previous coronary event with or without heart failure, or stroke). Moreover, among women we included age^2 instead of age in order to possibly adjust for menopausal status.

Potential interactions between gender and the each of the four biomarkers on incident fragility fractures were tested in the minimally adjusted models, with age and gender as covariates in addition to the biomarkers and the multiplicative interaction term [gender* levels of biomarker].

The proportional hazard assumption was tested by visual inspection of survival curves of quartiles of the biomarkers.

All analyses were performed using IBM SPSS Statistics version 24 (SPSS Inc., Chicago, IL, USA). All tests were two-sided whereby *p* < 0.05 was considered statistically significant.

## Results

### Baseline characteristics

Baseline data of the 5291 men and women that were included is shown in Table 1. The subjects were followed for a median time of 21.0 years (range, 1–23 years), during which 1002 subjects (18.9%) experienced at least one low-energy fracture. The median time from baseline to the first incident low-energy fracture was 14.5 years.

**Table 1:** Baseline characteristics of study population stratified by gender.

	All subjects (n = 5291)	Men (n = 2175)	Women (n = 3116)
Age, years	57.0 (5.9)	57.1 (6.0)	57.0 (5.9)
BMI, kg/m^2^	25.7 (4.0)	26.2 (3.5)	25.4 (4.2)
SBP, mmHg	141.4 (19.0)	143.4 (18.7)	140.0 (19.1)
Current smoker, %	26.7	27.8	26.0
Previous fracture, %	2.2	2.1	2.3
Prevalent CVD, %	2.3	4.0	1.1
AHT, %	16.9	17.9	16.2
**Biomarkers**			
MR-proADM, pm/L	0.46 (0.13)	0.46 (0.12)	0.47 (0.13)
MR-proANP, pm/L *	66.3 [35.7]	60.5 [32.9]	71.0 [35.0]
Copeptin, pm/L *	5.2 [5.1]	7.2 [6.0]	4.2 [3.7]
NT-pro-BNP, pm/L*	61.0 [78]	47.2 [69]	70.3 [82]

Results displayed as mean (SD) for continuous variables, unless otherwise specified

* Displayed as MD [interquartile range]. BMI = body mass index. SBP = systolic blood pressure. CVD = cardiovascular disease. AHT = antihypertensive treatment.

Subjects with incident fracture were older, more often women, had lower BMI and higher prevalence of previous fractures, whereas higher systolic blood pressure (SBP), anti-hypertensive medications and being a smoker were not associated with incident fracture during follow-up (Table 2). Levels of MR-pro-ADM, MR-pro-ANP, and NT-pro-BNP were slightly higher in subjects that experienced fractures during follow-up, whereas CT-pro-AVP were slightly lower (Table 2).

**Table 2:** Baseline characteristics of study population stratified by incident fracture during follow-up.

	All subjects (n = 5291)	No Fracture (n = 4289)	Fracture (n = 1002)	P-value**
Age, years	57.0 (5.9)	56.6 (5.9)	58.8 (5.7)	<0.001
Gender, % female	58.9	55.1	75.2	<0.001
BMI, kg/m^2^	25.7 (4.0)	25.8 (3.9)	25.2 (4.0)	<0.001
SBP, mmHg	141.4 (19.0)	141.2 (19.0)	142.3 (19.0)	0.107
Current smoker, %	26.7	26.8	26.3	0.733
Previous fracture, %	2.2	1.9	3.8	<0.001
Prevalent CVD, %	2.3	2.4	2.2	0.763
AHT, %	16.9	16.6	18.0	0.308
**Biomarkers**				
MR-proADM, pm/L	0.46 (0.13)	0.46 (0.13)	0.47 (0.15)	0.003
MR-proANP, pm/L *	66.3 [35.7]	65.3 [34.8]	70.4 [36.9]	<0.001
Copeptin, pm/L *	5.2 [5.1]	5.3 [5.1]	5.1 [5.2]	0.046
NT-pro-BNP, pm/L*	61.0 [78]	59.0 [78]	69.0 [85]	<0.001

Results displayed as mean (SD) for continuous variables, unless otherwise specified.

* Displayed as MD [interquartile range]

** P-value for independent samples T-test for continuous variables and Chi^2^ for dichotomous variables. Variables displayed as MD [interquartile range] were log-transformed in T-test analyses. BMI = body mass index. SBP = systolic blood pressure. CVD = cardiovascular disease. AHT = antihypertensive treatment.

### Baseline levels of cardiovascular biomarkers as predictors of incident fractures

**Fig 1:**
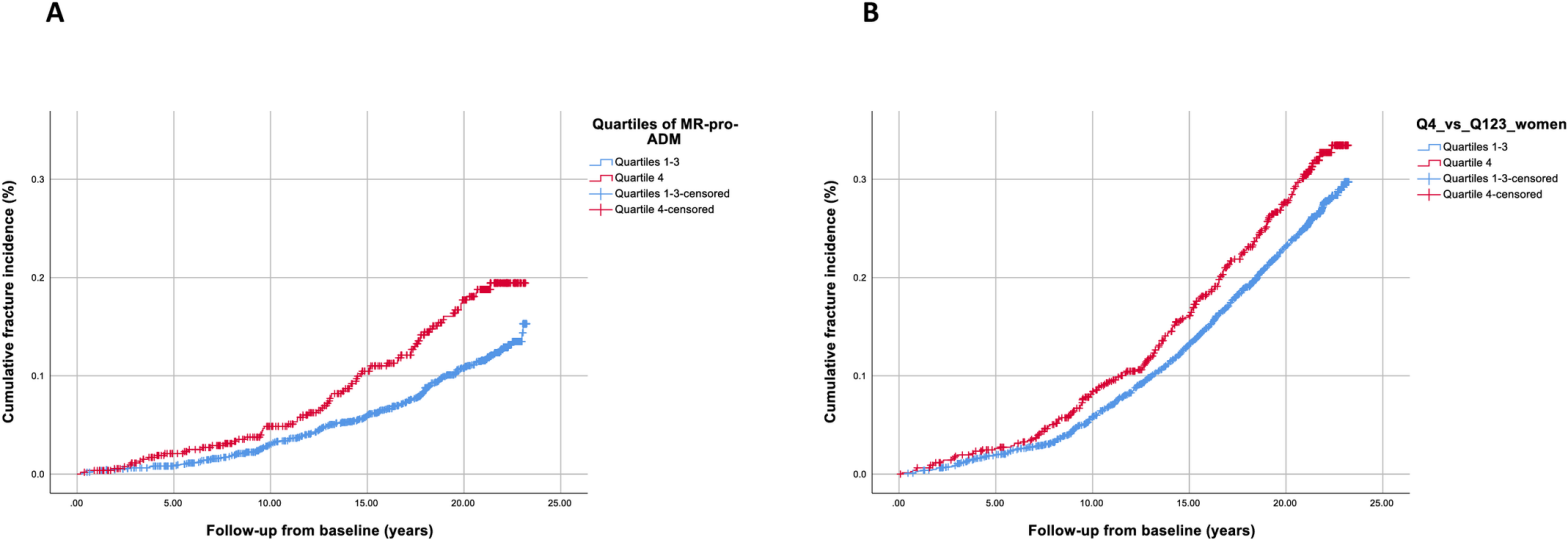
**Kaplan-Meier curves for incident fragility fractures among men (A) and women (B) stratified into highest quartile vs lower quartiles of mid-regional-pro-adrenomedullin plasma levels at baseline.** 4^th^ Quartile among men and women corresponded to ≥ 0.51 pm/L and ≥ 0.54 pm/L respectively.

There was no association between levels of MR-pro-ADM and incident low-energy fractures during follow-up among all subjects in age and gender-adjusted time-dependent models, however interaction analysis revealed a significant interaction between gender and levels of MR-pro-ADM on the risk of low-energy fractures during follow-up (p-interaction = 0.002). Accordingly, among men MR-pro-ADM significantly predicted incident fractures, whereas among women there was no such association (Table 3). Among men, the association between MR-pro-ADM levels and low-energy fractures remained significant in the multivariable adjusted model, including previous fracture. Kaplan-Meier curves for incident low-energy fracture according to the upper versus other quartiles of MR-pro-ADM among men and women respectively are displayed in Fig 1A and 1B.

**Table 3:** The association between MR-pro-ADM and incident fractures.

	Total events	HR per SD	95% CI	P-value
All subjects*	999	1.038	0.973–1.107	0.256
All subjects**	981	1.077	1.004–1.155	**0.039**
Men*	247	1.219	1.083–1.373	**0.001**
Men **	241	1.232	1.088–1.395	**0.001**
Women*	752	0.990	0.919–1.068	0.800
Women **	740	1.029	0.947–1.118	0.504

HR = hazard ratio. SD = standard deviation. See text for abbreviations of biomarkers.

* Adjusted for age and gender

** Adjusted for age, smoking, antihypertensive medication, systolic blood pressure, body mass index, previous low-energy-fracture, prevalent cardiovascular disease (coronary event or stroke), alcohol consumption (over or below median in the cohort). In women, age was substituted for age^2 in order to partly correct for menopausal status. P-interaction for [gender*MRADM] on incident low energy fracture = 0.002

Levels of MR-pro-ANP, CT-pro-AVP or NT-pro-BNP did not predict incident fragility fractures, nor were there any significant interactions between gender and levels of the hormones on incident fragility fracture (Table 4).

**Table 4:** The association between other biomarkers and incident fractures.

	Total events	HR per SD	95% CI	P-value
MR-pro-ANP (all)	999	0.998	0.932–1.069	0.998
CT-pro-AVP (all)	996	1.027	0.962–1.096	0.430
NT-pro-BNP (all)	974	1.034	0.965–1.108	0.338

HR = hazard ratio. SD = standard deviation. See text for abbreviations of biomarkers. Models adjusted for age and gender. P-interaction for [gender*MR-pro-ANP] on incident low energy fracture: 0.134; P-interaction for [gender*CT-pro-AVP] on incident low energy fracture: 0.140; P-interaction for [gender*NT-pro-BNP] on incident low energy fracture: 0.324.

As a sensitivity analysis we also performed all the analyses in subjects with complete data on all of the four biomarkers (n = 5122). The results were not substantially different from the main results (data not shown).

## Discussion

In this study, we demonstrated that levels of mid-regional-fragment of pro-adrenomedullin-peptide may predict fragility fractures among middle-aged men, but not among women. Even though MR-pro-ANP and NT-pro-BNP levels were slightly higher in subjects that experienced low-energy-fractures, these did not associate with low-energy fractures in our adjusted time-dependent Cox proportional hazard models.

Fractures due to low-energy trauma may be a valid surrogate for syncope or blood pressure instability (5) but may also demonstrate the comorbidity that exists between cardiovascular disease and low-energy fractures in an ageing population. There seems to be a strong correlation between cardiovascular instability, hypotension and traumatic falls. A review by Rubenstein et al concluded, that most common causes of falls were balance disorders, vertigo and dizziness[17]. Cardiovascular disorders[18], and syncope[19, 20] have also been associated with increased risks of falls. We have previously shown that orthostatic hypotension and higher heart rate at baseline are predictive of low-energy fractures in a large population based cohort[6]. Moreover, we have shown that some cardiovascular biomarkers, such as copeptin, MR-pro-ANP, C-terminal-pro-endothelin-1, and MR-pro-adrenomedullin may be altered in conditions usually related to unexplained falls and syncope[8, 9].

The positive finding between levels of MR-pro-ADM and low-energy-fractures deserves special consideration. MR-pro-ADM is a precursor fragment of neurohormone adrenomedullin, a 52 amino acid peptide with strong vasodilating properties. Several studies have shown that MR-proADM predicts coronary events: fatal or nonfatal myocardial infarction or death due to ischemic heart disease[11, 21]. The gender specific association between MR-pro-ADM and low-energy-fractures in our study is also interesting from a risk prediction perspective. Whereas low-energy-fractures are generally more common among women[4], the high mortality, especially in the first year, associated with these kinds of fractures is greater in men than in women[5, 22, 23].

Previous analyses of incidence rates of acute myocardial infarction by age and gender showed a well-known pattern of a higher incidence among men than among women, and a steep increase with age for both genders[24]. Thus, based on our current findings it could be tempting to hypothesize that MR-pro-ADM acts as a link between coronary artery disease and low-energy-fractures among men.

Moreover, plasma concentration of MR-proADM is significantly higher among patients with cardiac syncope (e.g. primary cardiac arrhythmia and structural heart disease) as well as in syncope due to orthostatic hypotension[25]. Increased MR-proADM levels were also found in patients with carotid sinus hypersensitivity, orthostatic hypotension and unexplained syncope after initial cardiovascular autonomic diagnostic workup was performed[8]. Thus, higher levels of MR-proADM may suggest presence of either cardiovascular autonomic dysfunction or susceptibility to paroxysmal arrhythmia with or without underlying structural heart disease as the potential cause of fall trauma. Taking into consideration the long-term follow-up and the median time from baseline to the first incident fracture of approximately 14 years, it seems likely that MR-pro-ADM predicted increased cardiovascular instability, and, consequently, higher risk of fall and bone fracture.

Above and beyond its cardiovascular effects, adrenomedullin has many other physiological functions such regulation of insulin secretion and influence on bone remodelling[26]. In an animal model, it has been observed that inhibition of adrenomedullin may prevent bone loss in ovariectomized mice[26]. A possible pathophysiological explanation of the role of increased adrenomedullin production in fragility fractures among middle-aged men should be sought in future studies.

Levels of MR-pro-ANP, CT-pro-AVP or NT-pro-BNP did not predict incident fragility fractures in this study, which is somewhat unexpected in the light of previous studies on these biomarkers. In the earlier studies, levels of MR-pro-ANP were markedly changed in common forms of syncope[8]. Moreover, levels of NT-proBNP were shown to be higher and predict coronary events in subjects undergoing emergency hip fracture surgery[27], however these biomarkers were analysed in the acute setting subsequent to the hip fracture. In a study by Chong et al[28], the pre- and postoperative levels of NT-proBNP did not predict 6-month mortality or cardiac events in a frail population of patients sustaining hip-fracture. However, also in this study, the cardiovascular biomarkers were assessed after the hip fracture had occurred, which is a different setting from our population-based design.

A potential clinical implication of our finding is the possibility that MR-pro-ADM may be used to improve clinical risk prediction of low-energy fractures. These fractures constitute a huge clinical problem, involving several specialities[29], often being a complicating factor in patients with several comorbidities and with a number of other known risk factors under treatment [30, 31].

Our study has a number of limitations that should to be mentioned. First, the authors had little influence on the data collected and original study design. Unfortunately, resting heart rate was not measured at baseline, in the MDC cohort, which would have been very informative for the current study. For the whole MDC cohort, cardiovascular biomarkers were only evaluated at baseline, indicating that we were unable to evaluate how any potential prospective change could be related to outcome. The main strengths of this study are the large number of subjects and reliability of collected data.

## Conclusion

Circulating levels of mid-regional-fragment of pro-adrenomedullin peptide predict fragility fractures among middle-aged men in the long-term, whereas levels of natriuretic peptides and copeptin are not associated, neither in males nor females, with these fractures. We propose further studies to understand the underlying mechanisms and potential use of MR-proADM as a risk predictor of bone fractures in men.

## Supporting information

S1 File A complete dataset of Malmö Diet and Cancer study (SPSS 24.0). (SAV)
